# Maternal Prebiotic Ingestion Increased the Number of Fecal Bifidobacteria in Pregnant Women but Not in Their Neonates Aged One Month

**DOI:** 10.3390/nu9030196

**Published:** 2017-02-26

**Authors:** Shinji Jinno, Takayuki Toshimitsu, Yoshitaka Nakamura, Takayuki Kubota, Yuka Igoshi, Naoko Ozawa, Shuichi Suzuki, Taiji Nakano, Yoshinori Morita, Takayasu Arima, Fumiya Yamaide, Yoichi Kohno, Kentaro Masuda, Naoki Shimojo

**Affiliations:** 1 Food Science Research Laboratories, R&D Division, Meiji Co., Ltd., 540 Naruda, Odawaara Kanagawa 250-0862, Japan; takayuki.toshimitsu@meiji.com (T.T.); yoshitaka.nakamura@meiji.com (Y.N.); 2 Department of Pediatrics, Graduate School of Medicine, Chiba University, Chiba 260-8670, Japan; takayuki.kubota@meiji.com (T.K.); yuka_nz_kumamoto@hotmail.com (Y.I.); n.ozawa0904@gmail.com (N.O.); taibokku2000@yahoo.co.jp (T.N.); m0ritan@hotmail.com (Y.M.); takayasuarima@gmail.com (T.A.); fyamaide@chiba-u.jp (F.Y.); kohnoy@chibah.rofuku.go.jp (Y.K.); shimojo@faculty.chiba-u.jp (N.S.); 3 Department of Pediatrics, National Shimoshizu Hospital, Chiba 284-0003, Japan; seeyou@msj.biglobe.ne.jp; 4 Masuda Maternity Clinic, Chiba 289-2144, Japan; ke-masuda@umin.ac.jp

**Keywords:** fructooligosaccharides, bifidobacteria, feces, infancy, pregnancy, prebiotic, constipation, stool frequency

## Abstract

Fructooligosaccharides (FOS) can selectively stimulate the growth of bifidobacteria. Here, we investigated the effect of maternal FOS ingestion on maternal and neonatal gut bifidobacteria. In a randomized, double-blind, placebo-controlled study, we administered 8 g/day of FOS or sucrose to 84 women from the 26th week of gestation to one month after delivery. The bifidobacteria count was detected using quantitative PCR in maternal (26 and 36 weeks of gestation) and neonatal (one month after delivery) stools. Maternal stool frequency was recorded from 24 to 36 weeks of gestation. The number of fecal *Bifidobacterium* spp. and *Bifidobacterium longum* in the FOS group was significantly higher than that in the placebo group at 36 weeks of gestation (2.7 × 10^10^/g vs. 1.1 × 10^10^/g and 2.3 × 10^10^/g vs. 9.7 × 10^9^/g). In their neonates, these numbers did not differ between the groups. Also, stool frequency in the FOS group was slightly higher than that in the placebo group two weeks after the intervention (1.0 vs. 0.8 times/day), suggesting a potential constipation alleviation effect. In conclusion, the maternal FOS ingestion showed a bifidogenic effect in pregnant women but not in their neonates.

## 1. Introduction

Bifidobacteria are the predominant commensal bacteria in neonates, and they colonize the neonatal gut immediately after birth [1]. Their colonization after birth occurs through the vertical transfer of gut microbiota from the mother to the neonate [2,3] and is influenced by several factors [4] such as gestational age at birth [5], the mode of delivery [6], use of perinatal antibiotics [7], or the mode of feeding [8]. Recent studies have shown that one of the main sources of bifidobacteria is breast milk [9,10,11]. This gut microbiota influences host nutrition, provides a natural defense mechanism against invading pathogenic bacteria [12], and may determine the development of the infant’s immune system [13,14]. Consequently, previous research has aimed to develop mechanisms to modify the composition of the neonatal gut microbiota, including the oral administration of prebiotics to pregnant women [15].

Fructooligosaccharides (FOS) are a type of prebiotics composed of 1-kestose, nystose, and 1F-ß-fructofuranosylnystose [16]. After ingestion, they enhance the growth of gut bifidobacteria in animals and humans [16]. In pregnant women, FOS ingestion alleviates constipation [17]; however, little is known about the effect of maternal FOS ingestion on maternal and neonatal gut bifidobacteria. 

Here, we hypothesized that maternal FOS ingestion increases the number of bifidobacteria in the maternal gut, and these bifidobacteria can be vertically transferred from the mother to the neonate. Thus, we conducted a double-blind, randomized, placebo-controlled study to investigate the effect of maternal FOS ingestion on the number of gut bifidobacteria in both the maternal and neonatal gut. To evaluate the bifidobacterial transfer in detail, we focused on *Bifidobacterium longum* (*B. longum*) because it is the main species that inhabits both the infant and adult gut [1] and can multiply in both the maternal and neonatal gut by utilizing FOS or Human milk oligosaccharides (HMOs) [18,19]. In maternal feces, we also detected predominant bacteria present in adults, such as the *Clostridium coccoides* group, *Clostridium leptum* subgroup, *Bacteroides* spp., and *Enterobacteriaceae* [1,20,21], to monitor the prebiotic effect on the microbiota other than that of bifidobacteria. In addition, we evaluated whether FOS ingestion influences stool characteristics and the frequency of adverse events in pregnant women.

## 2. Materials and Methods

### 2.1. Subjects and Study Protocol

**Figure 1:**
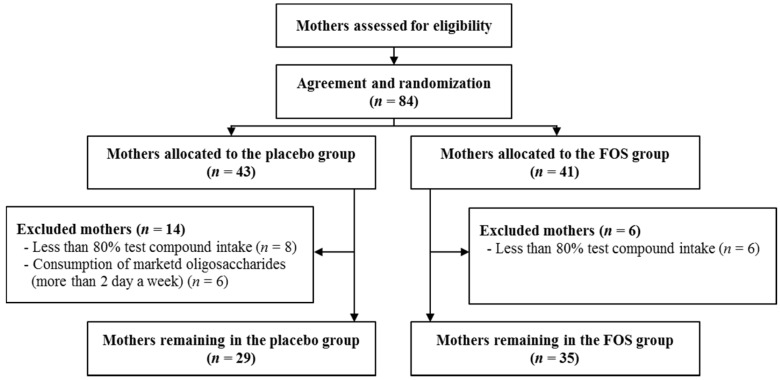
Diagram showing the flow of participants in the placebo and FOS groups.

The present randomized, double-blind trial was registered in the UMIN Clinical Trial Registry (UMIN000008142) [22]. This trial was conducted at the Masuda Maternity Clinic in Chiba prefecture. Healthy pregnant woman were enrolled after they provided written informed consent from September 2009 to December 2011 [23]. Pregnant women with complications such as pregnancy-induced hypertension were excluded, although subjects with a history of allergic disease were included. The study was conducted according to the guidelines of the Declaration of Helsinki, and all the procedures were approved by the Ethics Committee of Chiba University. Subjects (84 pregnant women) were randomly assigned to the FOS (Meioligo-P, from Meiji Food Materia Company Limited, Tokyo, Japan) (FOS, *n* = 41) or sucrose (placebo, *n* = 43) intake groups (Figure 1). We administered 8 g of the trial compound as an oral daily dose between the 26th week of gestation and one month after delivery. The trial compound was divided into two 4 g doses. A 4 g dose was administered in each group twice daily. We did not impose a limit on the daily ingestion of oligosaccharides to the study subjects for ethical reasons. However, based on their diaries and food frequency questionnaire results, we excluded those subjects from the placebo group who ingest marketed oligosaccharide products for more than two days a week. In addition, we excluded those subjects from both groups with less than 80% test compound intake due to insufficient intervention (Figure 1).

### 2.2. Fecal Sample Collection

We collected two maternal stool samples from each subject immediately before the first treatment (at 26 weeks of gestation) and 10 weeks after the start of the treatment (at 36 weeks of gestation). In addition, we collected one neonatal stool sample per infant at one month of age. The mothers were asked to freeze the samples immediately after collection. The samples were subsequently kept frozen during transport and stored at −20 °C until further analyses.

### 2.3. Analysis of Fecal Microbiota

Bacterial DNA was extracted from the stool samples using a QIAamp stool mini kit (QIAGEN; Valencia, CA, USA). Briefly, 20 mg of stool samples were disrupted in 700 μL of ASL Buffer with zirconia beads (0.1 and 0.3 mm) using a tissue lyser at a frequency of 25.0 Hz for 15 min. The samples were subsequently incubated at 70 °C for 15 min. Impurities and PCR inhibitors were removed by adding InhibitEX Tablets to each sample. The DNA in the supernatants was then purified using QIAamp mini-spin columns. This procedure included protein digestion, a wash to remove any impurities, and an elution step to obtain a purified DNA sample. We used quantitative real-time polymerase chain reaction (q-RT-PCR) to quantify the number of gut microbiota using locus-specific primers, as previously described [21,24]. The primer sequences were as follows: *Bifidobacterium* spp. sense primer: 5′-CTCCTGGAAACGGGTGG-3′; *Bifidobacterium* spp. antisense primer: 5′-GGTGTTCTTCCCGATATCTACA-3′; *Bacteroides* spp. sense primer: 5′-ATAGCCTTTCGAAAGRAAGAT-3′; *Bacteroides* spp. antisense primer: 5′-CCAGTATCAACTGCAATTTTA-3′. *Enterobacteriaceae* sense primer: 5′-CATTGACGTTACCCGCAGAAGAAGC-3′; *Enterobacteriaceae* antisense primer: 5′-CTCTACGAGACTCAAGCTTGC-3′; *Clostridium coccoides* group sense primer: 5′-AAATGACGGTACCTGACTAA-3′; *Clostridium coccoides* group antisense primer: 5′-CTTTGAGTTTCATTCTTGCGAA-3′; *Clostridium leptum* subgroup sense primer: 5′-GCACAAGCAGTGGAGT-3′; *Clostridium leptum* subgroup antisense primer: 5′-CTTCCTCCGTTTTGTCAA-3′; and *Bifidobacterium longum* (*B. longum*) sense primer: 5′-TTCCAGTTGATCGCATGGTC-3′; *Bifidobacterium longum* antisense primer: 5′-GGGAAGCCGTATCTCTACGA-3′; Each PCR reaction mixture contained 22.5 pmol of each primer, 12.5 μL of 2× QuantiFast SYBR Green PCR Master Mix (QIAGEN; Valencia, CA, USA), and a DNA template in a total volume of 25 μL. To quantify the number of *Bifidobacterium* spp. in maternal and neonatal guts, we used a PCR amplification protocol comprising an initial denaturation step for 15 min at 95 °C followed by 45 cycles of melting, annealing, and extension at 94 °C for 15 s, 60 °C for 30 s, and 72 °C for 30 s, respectively. To quantify *Bacteroides* spp. and *Clostridium leptum* subgroup numbers, we used the following PCR amplification protocol: initial denaturation for 15 min at 95 °C followed by 45 cycles of melting, annealing, and extension at 94 °C for 15 s, 50 °C for 30 s, and 72 °C for 30 s, respectively. To quantify the Enterobacteriaceae number, we used the following PCR amplification protocol: initial denaturation for 15 min at 95 °C followed by 45 cycles of melting, annealing, and extension at 94 °C for 15 s, 58 °C for 30 s, and 72 °C for 30 s, respectively. To quantify the *Clostridium coccoides* number, we used the following PCR amplification protocol: initial denaturation for 15 min at 95 °C followed by 45 cycles of melting, annealing, and extension at 94 °C for 15 s, 50 °C for 30 s, and 72 °C for 45 s, respectively. To quantify the *B. longum* number, we used the following PCR amplification protocol: initial denaturation for 15 min at 95 °C followed by 45 cycles of melting, annealing, and extension at 94 °C for 15 s, 55 °C for 30 s, and 72 °C for 30 s, respectively. Calibration curves for quantification were performed with genomic DNA isolated from a known number of each species of bacteria.

### 2.4. Stool Characteristics and Delivery and Feeding Modes

At 24, 28, 32, and 36 weeks of gestation, we collected daily data on stool consistency and frequency, diarrhea, and abdominal pain. The outcome parameters were stool frequency per day, stool consistency according to the Bristol Stool Form Scale (stools are rated based on the water content of the feces on a scale from 1 to 7, with 1 meaning hard stools and 7 meaning liquid stools) [25], frequency of abdominal pain per week, and frequency of diarrhea per week. The mode of delivery was recorded in the hospital. The mode of feeding (i.e., breast-feeding or formula-feeding) was reported by the mothers.

### 2.5. Exclusion from the Analyses

In the analysis of maternal feces, we excluded one subject from the FOS group because we did not receive her stool samples. In the analysis of the neonatal feces, we excluded those neonates born by cesarean delivery (C-section) were excluded (*n* = 2 and *n* = 3 for the placebo and FOS group, respectively) because the mode of delivery influences the compositon of neonatal microbiota [26,27]. We also excluded two neonates from the placebo group because we did not receive their stool samples. In the analysis of *B. longum* of neonatal feces, one neonate was additionally excluded because of the absence of the sample. Finally, in the stool characteristic and adverse event analyses, we excluded those mothers who did provide with less than four daily records per week. 

### 2.6. Statistics

The values are expressed as medians or means, depending on their distribution. We compared the differences between groups using Student’s *t*-test for parametric data, Mann-Whitney *U* test for nonparametric data and chi-squared tests for categorical data. To determine the relation between the number of fecal bifidobacteria in mothers and neonates, we used the Spearman’s rank correlation coefficient. For all tests, *p* < 0.05 was considered statistically significant. The statistical analyses were performed using the Bell Curve for Excel (Social Survey Research Information Co., Ltd., Tokyo, Japan). 

## 3. Results

After exclusions, we included 29 subjects in the placebo group and 35 in the FOS group (Figure 1). As previously reported, no significant differences were observed in the characteristics and dietary habits of the subjects between the groups (Table 1) [23]. 

**Figure 2:**
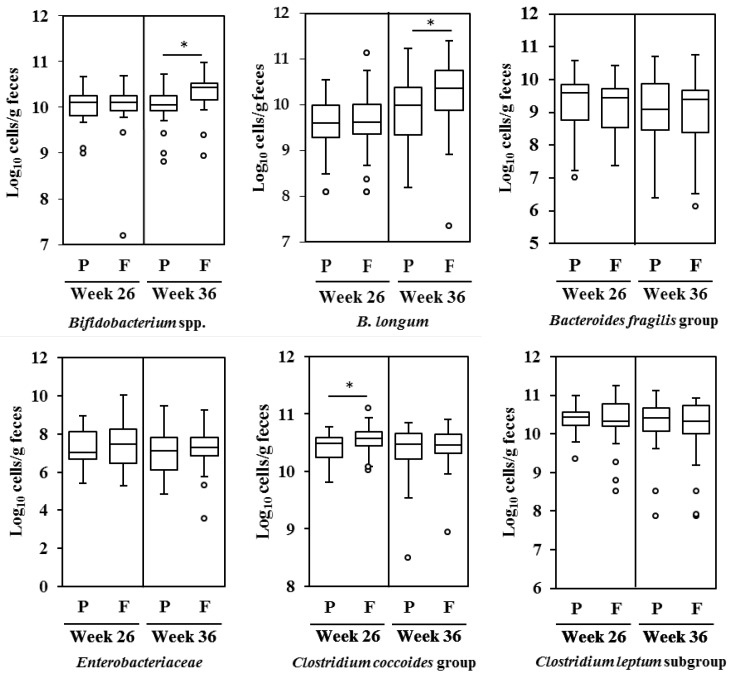
The number of gut microbiota, expressed as the logarithm of cell numbers, in maternal feces at 26 and 36 weeks of gestation. Microbiota were quantified using q-RT-PCR (*n* = 29 and 34 for the placebo and FOS group, respectively). Box and whiskers plot, with the black horizontal line representing the median value and the boxes the interquartile ranges. The T-bars represent the data range, and the open circles indicate the presence of outliers (data points more than 1.5 interquartile ranges below the first quartile or above the third quartile). * Statistically different based on a Mann-Whitney *U* test (*p* < 0.05). P: Placebo group; F: FOS group.

Figure 2 shows the estimated number of *Bifidobacterium* spp., *B. longum*, the *Bacteroides fragilis* group, *Enterobacteriaceae*, the *Clostridium coccoides* group, and the *Clostridium leptum* subgroup in maternal feces at 26 and 36 weeks of gestation (before intervention and 10 weeks after intervention, respectively), based on q-RT-PCR data. The number of fecal *Bifidobacterium* spp. in the FOS group at 36 weeks of gestation (2.7 × 10^10^/g) was significantly higher than that in the placebo group (1.1 × 10^10^/g, Mann-Whitney *U* test, *p* < 0.05, Figure 2). Also, the number of fecal *B. longum* in the FOS group at 36 weeks of gestation (2.3 × 10^10^/g) was significantly higher than that in the placebo group (9.7 × 10^9^/g, Mann-Whitney *U* test, *p* < 0.05, Figure 2). The number of fecal *Clostridium coccoides* before the intervention was also higher in the FOS group than that in the placebo group (Mann-Whitney *U* test, *p* < 0.05, Figure 2).

**Figure 3:**
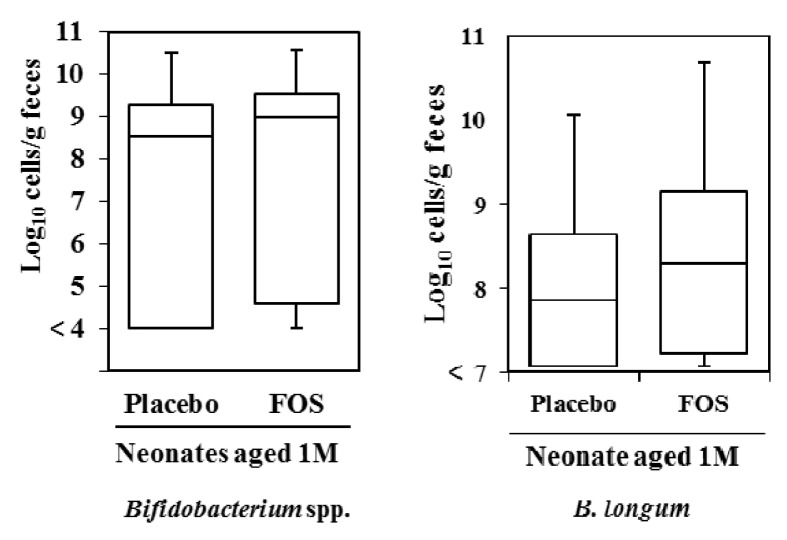
Infant fecal *Bifidobacterium* spp. and *B. longum* at one month of age, excluding neonates born by C-section. The number of *Bifidobacterium* spp. was quantified using q-RT-PCR (*n* = 25 and 30 in the analysis of *B. longum*) for the placebo and FOS group, respectively). Box and whiskers plot, with the black horizontal line representing the median value and the boxes the interquartile ranges. The T-bars represent the data range, and the open circles indicate the presence of outliers (data points more than 1.5 interquartile ranges below the first quartile or above the third quartile). No significant difference was observed between the groups (Mann-Whitney *U* test).

**Figure 4:**
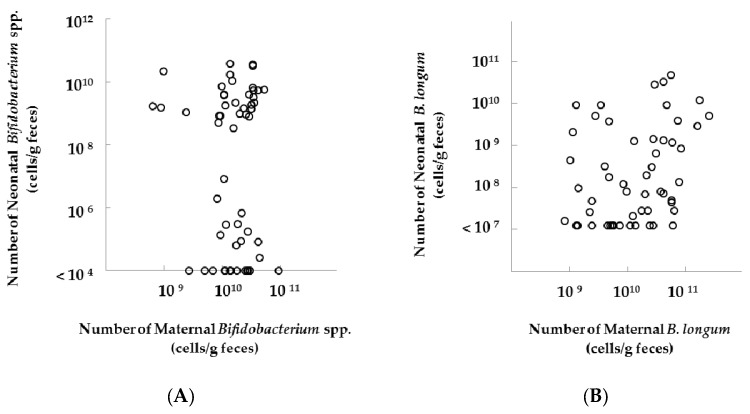
Relations between the number of fecal (**A**) *Bifidobacterium* spp. or (**B**) *B. longum* in mothers (at 36 weeks of gestation) and their neonates (one month after delivery), excluding neonates born by C-section. The number of *Bifidobacterium* spp. was quantified using q-RT-PCR (*n* = 25 and 31 in placebo and FOS group, respectively). Data were analyzed using the Spearman’s rank correlation coefficient ((**A**): *ρ* = 0.09, *p* = 0.50; (**B**): *ρ* = 0.33, *p* = 0.01).

In the analyses of neonatal feces, we did not observe any differences in the number of *Bifidobacterium* spp. and *B. longum* in neonates at one month of age between groups, after excluding neonates born by C-section (Figure 3). Additionally, we found no correlation in the number of fecal *Bifidobacterium* spp. (*ρ* = 0.09, *p* = 0.50, Figure 4) and a significant correlation in the number of fecal *B. longum* (*ρ* = 0.33, *p* = 0.01, Figure 4) between mothers and their neonates (Spearman’s rank correlation coefficient, Figure 4). In the subgroup analysis, the significant positive correlation in the fecal number of *B. longum* was observed between mothers and their neonates in the FOS group (Spearman’s rank correlation coefficient, *ρ* = 0.52, *p* < 0.05, Table 2).

**Figure A1:**
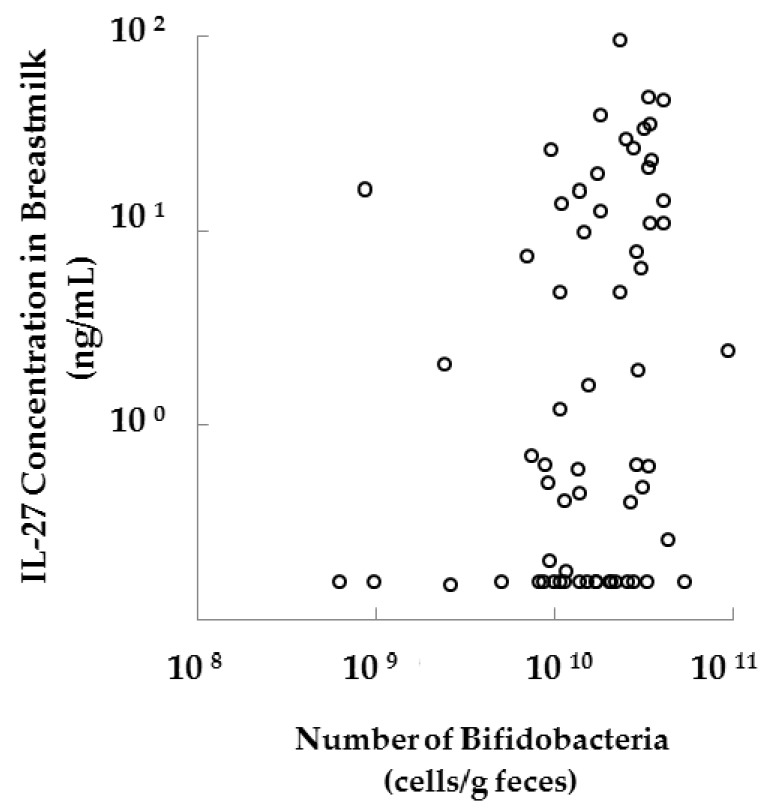
A significant correlation between the fecal bifidobacterial count in mothers and IL-27 concentration in their breast milk. The number of *Bifidobacterium* spp. was quantified by q-RT-PCR (*n* = 29 and 34 for the placebo and FOS group, respectively). IL-27 in colostrum whey was detected using The Human IL-27 DuoSet ELISA kit (R&D Systems). Data were analyzed using the Spearman’s rank correlation coefficient (*p* < 0.05).

We did not find any significant difference between groups regarding the stool form score, frequency of abdominal pain, and frequency of diarrhea two weeks before the intervention or two, six, and 10 weeks after the intervention (at 24, 28, 32, 36 weeks of gestation, respectively) (Table 3). Stool frequency in the FOS group was significantly higher only two weeks after the intervention (28 weeks of gestation) than in the placebo group (1.0 vs. 0.8 times per day, Mann-Whitney *U* test, *p* < 0.05). In addition, a significant correlation was observed between the fecal bifidobacterial count in mothers and the interleukin-27 (IL-27) concentration in their breast milk (IL-27 concentration was shown in our previous report [23]) (Spearman’s rank correlation coefficient, *p* < 0.05, Figure A1).

## 4. Discussion

The present clinical trial investigated the effects of maternal prebiotic ingestion on maternal and neonatal gut bifidobacteria. Here, we show that the daily FOS intake (8 g/day) increased the number of fecal bifidobacteria in pregnant women, consistent with previous reports describing FOS bifidogenic activity at doses ranging from 2.5 to 10 g/day in healthy humans [28]. However, there was no difference in the number of fecal bifidobacteria between neonates whose mothers received FOS and those whose mothers did not take FOS (control). Also, stool frequency in the FOS group was slightly higher than that in the placebo group two weeks after the intervention (1.0 vs. 0.8 times/day), suggesting a potential constipation alleviation effect. 

The present study showed that the FOS-mediated increase in maternal fecal bifidobacteria did not affect the number of *Bifidobacterium* spp. in the feces of neonates aged one month. These results are also supported by a previous study on maternal supplementation with another prebiotic (galactooligosaccharides/long-chain FOS) [15], which showed that the proportion of bifidobacteria found in neonatal feces did not differ between the placebo and the supplemented groups. In addition, we find no significant correlations between the number of fecal *Bifidobacterium* spp. in mothers and their neonates, which is also consistent with previous non-interventional studies [3,29]. There are some aspects that could explain these results. First, the effect of maternal FOS ingestion in neonates might appear earlier (within the first month after birth). Second, maternal FOS ingestion might not lead to a significant increase in the number of vaginal bifidobacteria to be detected as transferred to the neonate because dietary FOS cannot reach the vagina. Third, the bifidobacterial species that were transferred to infants could not increase in the individual intestinal microbiome because neonatal stable engraftment of bifidobacteria was shown to depend on the individual microbiome [30]. Fourth, if the bifidobacterial species that were transferred to infants were not genetically capable of utilizing or degrading HMOs in breast milk, then the neonatal bifidobacterial number would not increase. In fact, the abundance of fecal bifidobacteria in neonates was shown to be greatly influenced by the utilization of HMOs [18]. To consider the fourth aspect, we quantified the number of *B. longum* in the maternal and neonatal feces. Here, maternal FOS ingestion resulted in high levels of *B. longum* in the mothers but not in their neonates, although it is capable of utilizing HMOs [18]. Thus, we found no evidence of a bifidogenic effect on the neonates by maternal FOS ingestion.

In the present study, 30 of 64 infants were exclusively breastfed at one month (Table 1). This low percentage for exclusive breastfeeding may have influenced the low correlation between maternal and infant fecal bifidobacteria. To clarify the effect of breastfeeding on the bifidobacterial transfer, we performed subgroup analyses on the number of *Bifidobacterium* spp. in exclusively breastfed infants of each group. Then, we did not find any significant correlation in exclusively breastfeeding pairs (Table 2). Meanwhile, the correlation seems to be higher in exclusively breastfed infants than in all infants. Interestingly, this correlation was low in the FOS group. It might be because bifidobacteria species that can utilize FOS but not HMOs increased in the maternal feces of the FOS group. Further research is required to investigate the effect of breastfeeding on the bacterial transfer. 

The present study showed that FOS was well tolerated by pregnant women at a dose of 8 g/day. Previous clinical trials assessing an FOS intake of ≤10 g/day have been reported for adult women [31,32,33,34,35]; however, the number of reports on FOS perinatal ingestion in women has reduced [17]. In the present study, we found no differences in the frequency of abdominal symptoms in the FOS group compared with those in the placebo group until 10 weeks after the start of the intervention. Additionally, the maternal FOS intake (8 g/day) was shown to increase the number of fecal bifidobacteria. These findings suggested that the ingested dose of FOS (8 g/day) was appropriate for pregnant women.

Our results suggest a potential constipation alleviation effect of FOS in pregnant women. The prevalence of constipation in pregnancy ranges from 11% to 44% [36] and has been associated with a decrease in the quality of life for pregnant women. Dietary supplementation composed of FOS, *Lactobacillus*, and *Bifidobacterium* has been shown to increase stool frequency in constipated adult women [32]. A similar effect has been observed for probiotics [37] or FOS [38]. However, little is known about the effect of FOS on stool frequency in pregnant women [17]. The increased stool frequency observed in our results suggested that dietary FOS helps alleviate the gastrointestinal discomfort in pregnant women. Further research in pregnant women with constipation should investigate the effect of FOS ingestion on constipation during pregnancy.

Maternal prebiotic consumption may also affect immunoactive constituents in breast milk by altering the gut microbiota. In a previous report on this clinical trial, we showed that the IL-27 concentration in breast milk increased in the FOS group compared with that in the placebo group [23]. A further analysis also showed a significant correlation between the IL-27 concentration in breast milk and bifidobacterial counts in maternal feces (Figure A1; the Spearman’s rank correlation coefficient, *p* < 0.05). Dietary FOS has been shown to increase the number of gut *Bifidobacterium* spp. and *Bacteroides* spp. as well as the total IgA concentration in both the intestine and milk in mice, and the IgA concentration in milk was correlated with the number of gut *Bacteroides* spp. [39]. In the present study, however, the IgA concentration in the colostrum did not differ between the groups [23]. A possible cause is the presence of a large individual diversity in the gut microbiota that utilize prebiotics. In fact, the IgA concentration did not change in response to dietary FOS in some subjects [40]. Thus, the choice of prebiotics may be important to provide the appropriate immunomodulating effect in neonates through breast milk.

There are some limitations of this study. First, we did not control the daily consumption of oligosaccharides in pregnant women for ethical reasons. Consequently, we excluded those subjects who consumed marketed oligosaccharide products for more than two days a week from the placebo group. Thus, the increases in the number of fecal bifidobacteria and stool frequency found in the FOS group might partly result from the consumption of other oligosaccharides. Second, we quantified the number of bacteria by using q-RT-PCR. A 16S rRNA gene-based metagenomic analysis would add a great deal to the study results.

## 5. Conclusions

In conclusion, the present study showed a bifidogenic effect of FOS in pregnant women at a dose of 8 g/d with no evidence of adverse effects. Also, no evidence was obtained about a bifidogenic effect on the neonates by maternal FOS ingestion.

## Figures and Tables

**Table 1:** Characteristics of the mothers and the neonates.

	Placebo Group *n* = 29	FOS Group *n* = 35	*p*
**Mothers’ characteristics**			
Age (median, years)	33	30	N.S.*
Primipara (*n*)	13	16	N.S. †
Pollen disease (*n*)	8	10	N.S. †
History of atopic diseases (*n*)	1	6	N.S. †
History of other allergic diseases (*n*)	10	20	N.S. †
Smoking during pregnancy (*n*)	6	3	N.S. †
BMI during prepregnancy (median)	21.2	20.3	N.S. *
BMI during pregnancy (median)	25.3	24.6	N.S. *
**Neonates’ characteristics**			
Fetus week number (median, weeks)	39.1	39.3	N.S. *
Birth weight (median, g)	2990	3165	N.S. *
Caesarean section (*n*)	2	3	N.S. †
Exclusively breastfed (*n*)	14	16	N.S. †
Exclusively formula-fed (*n*)	0	0	N.S. †

FOS, fructo-oligosaccharides. BMI, body mass index. * Mann–Whitney U-test. † Pearson’s x2 test.

**Table 2:** Subgroup analysis for relation between the number of fecal bifidobacteria in mothers and their neonates.

Subgroup		*Bifidobacterium* spp.			*B. longum*		
		*n*	*ρ*	*p*	*n*	*ρ*	*p*
**Placebo-group**	All infants	25	0.19	0.37	25	−0.22	0.25
	Exclusively breastfed infants	13	0.41	0.16	13	0.14	0.64
**FOS-group**	All infants	31	−0.02	0.94	30	0.49	0.01
	Exclusively breastfed infants	16	0.15	0.56	16	0.40	0.17

**Table 3:** Stool characteristics and adverse events recorded in pregnant women two weeks before the intervention and two, six, and 10 weeks after the intervention.

**Weeks of Gestation**	**24**		**28**		**32**		**36**	
**Weeks from the Start of Supplementation**	**−2**		**2**		**6**		**10**	
**Group**	***P***	***F***	***P***	***F***	***P***	***F***	***P***	***F***
*n*	25	27	26	31	24	33	25	32
Stool frequency (times per day), median	0.9	1.0	0.8	1.0 *	1.1	1.0	1.2	1.0
Stool form score, mean	3.9	4.2	3.9	4.2	3.9	4.0	3.9	4.3
Frequency of abdominal pain (days per week), median	0	0	0	0	0	0	0	0
frequency of diarrhea (days per week), median	0	0	0	0	0	0	0	0

*P* = Placebo-group, *F* = Fructooligosaccharides-group. * *P* < 0.05 (Mann-Whitney *U* test).
